# Network Pharmacology-Based Prediction and Verification of Ginsenoside Rh2-Induced Apoptosis of A549 Cells *via* the PI3K/Akt Pathway

**DOI:** 10.3389/fphar.2022.878937

**Published:** 2022-05-04

**Authors:** Chao Song, Yue Yuan, Jing Zhou, Ziliang He, Yeye Hu, Yuan Xie, Nan Liu, Lei Wu, Ji Zhang

**Affiliations:** ^1^ Jiangsu Collaborative Innovation Center of Regional Modern Agriculture and Environmental Protection, School of Life Sciences, Huaiyin Normal University, Huaian, China; ^2^ School of Pharmaceutical Sciences, Institute for Chinese Materia Medica, Tsinghua University, Beijing, China; ^3^ Beijing Increasepharm Safety and Efficacy Co., Ltd, Beijing, China; ^4^ Institute of Applied Chemistry, Academy of Sciences, Nanchang, China

**Keywords:** ginsenoside Rh2, network pharmacology, lung cancer, A549 cells, PI3K-Akt signaling pathway

## Abstract

Ginsenoside Rh2 (G-Rh2), a rare protopanaxadiol (PPD)-type triterpene saponin, from *Panax ginseng* has anti-proliferation, anti-invasion, and anti-metastatic activity. However, the mechanisms by which G-Rh2 induces apoptosis of lung cancer cells are unclear. In the present work, a G-Rh2 target-lung cancer network was constructed and analyzed by the network pharmacology approach. A total of 91 compound-targets of G-Rh2 was obtained based on the compound-target network analysis, and 217 targets were identified for G-Rh2 against lung cancer by PPI network analysis. The 217 targets were significantly enriched in 103 GO terms with FDR <0.05 as threshold in the GO enrichment analysis. In KEGG pathway enrichment analysis, all the candidate targets were significantly enriched in 143 pathways, among of which PI3K-Akt signaling pathway was identified as one of the top enriched pathway. Besides, G-Rh2 induced apoptosis in human lung epithelial (A549) cells was verified in this work. G-Rh2 significantly inhibited the proliferation of A549 cells in a dose-dependent manner, and the apoptosis rate significantly increased from 4.4% to 78.7% using flow cytometry. Western blot analysis revealed that the phosphorylation levels of p85, PDK1, Akt and IκBα were significantly suppressed by G-Rh2. All the experimental findings were consistent with the network pharmacology results. Research findings in this work will provide potential therapeutic value for further mechanism investigations.

## Introduction

Lung cancer is one of the most diagnosed cancers and a leading cause of cancer deaths. Smoking is the main cause (∼80% of cases) of lung cancer (Huang et al., 2021). Other causes are exposure to radon, secondhand smoke, and air pollution. In 2020, lung cancer was the second most common human cancer globally, there were 2,206,771 new cases (11.4% of all cases). The number of new lung cancer deaths was 17,966,144, accounting for 18% of all cancer deaths (Wild et al., 2020). Lung cancer can be divided into non-small cell lung cancer (NSCLC) and small cell lung cancer (SCLC) (Hanna and Onaitis, 2013). Treatment for lung cancer differs according to subtype and stage (Hayashi et al., 2011). Chemotherapy and radiotherapy have side effects (Albano et al., 2021), and even targeted immunotherapeutic have a considerable symptom burden (Lim et al., 2020). Therefore, research on new drugs and combined therapies is needed.

Ginseng, a traditional Chinese herb, has been used as medicine for thousands of years. It is used in cancer treatment and prevention based on its multi-target activity and low toxicity. Ginsenosides are the main active constituents in ginseng. G-Rh2, a 20 (S)-protopanaxadiol saponin extracted from the root of *Panax ginseng* (Wong et al., 2015), has been reported to show cytotoxic activity and decreased cancer cells viability *via* JAK2/STAT3 pathway in human colorectal cancer cells (Han et al., 2016), stimulates ROS production in human HeLa cervical cancer cell lines (Liu et al., 2021). G-Rh2 was also reported with antitumor effects in liver, lung, prostate, and colorectal cancer (Ge et al., 2017; Shi et al., 2017; Wu et al., 2018; Zhang et al., 2021). G-Rh2 inhibits proliferation, metastasis, and apoptosis by activating the mitochondrial or membrane death receptor (Wang et al., 2017). However, the molecular targets and signaling pathways underlying the effect of G-Rh2 on lung cancer are unclear. Cancer is typically caused by multiple genes and risk factors, so identification of multiple targets is important for understanding the mechanisms underlying the effect of G-Rh2 on lung cancer.

Network pharmacology is a systematic approach that integrates pharmacologic, computational, and experimental methods to illuminate the molecular mechanisms of drugs (Zhao and He, 2018; Song et al., 2019; Zhou et al., 2020). It can describe the complex pharmacological mechanisms of traditional Chinese medicines from a network perspective by multitarget, multichannel, and multilink analysis (Li et al., 2015; Park et al., 2018). In this work, we used the network pharmacology approach to evaluate the molecular mechanisms underlying the effect of G-Rh2 in lung cancer.

## Materials and Methods

### Materials and Reagents

**FIGURE 1:**
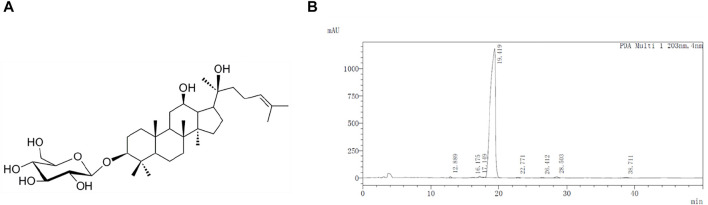
Molecular structure **(A)** and HPLC profile **(B)** of G-Rh2.

G-Rh2 was purchased from Chengdu Phytoelite Bio-Technology Co., Ltd. (Chengdu, Sichuan, China) and the purity (>98%) was determined by high-performance liquid chromatography (Figure 1). 3-(4,5-Dimethylthiazol-2-yl)-2,5-diphenyltetrazolium bromide (MTT) and Annexin V-FITC apoptosis detection kit were purchased from Sigma-Alorich (St. Louis, MO, United States). The fetal bovine serum (FBS) was purchased from Corning (Medford, MA, United States). Antibiotics (100× penicillin/streptomycin) and 0.25% Trypsin-EDTA were purchased from Gibco (California, United States). Minimum essential medium (MEM) was purchased from Hyclone (Logan, UT, United States). RIPA Lysis Buffer and eECL Western Blot kit were purchased from CWBio (Taizhou, Jiangsu, China). The primary antibodies against p-AKT, AKT, p-PDK1, PDK1, p-p85, p85, p-IκBα, IκBα, and anti-rabbit IgG HRP (#7074) were from Cell Signaling Technology (Danvers, MA, United States).

### Potential Targets Screening

The chemical structure of G-Rh2 was imported into PharmMapper server (http://www.lilab-ecust.cn/pharmmapper/, version 2017) (Xia et al., 2017), STITCH database http://stitch.embl.de/, version 5.0) (Szklarczyk et al., 2016), SwissTargetPrediction (http://www.swisstargetprediction.ch/) (David et al., 2014), and Similarity ensemble approach (http://sea.bkslab.org/) (Keiser et al., 2007) to obtained the related targets of G-Rh2.

### Lung Cancer-Related Targets Screening

The potential targets of Lung cancer were identified by the Genetic Association Database (https://geneticassociationdb.nih.gov/), which is a database of genetic association data from complex diseases and disorders. Lung cancer was imported as a keyword and the disease targets associated with it were provided in the database.

### Network Construction

In order to explore the relationship between G-Rh2 related targets and lung cancer disease related targets, protein-protein interaction (PPI) were analysed by the Database of Interacting Proteins (DIP^TM^), Biological General Repository for Interaction Datasets (BioGRID), Human Protein Reference Database (HPRD), IntAct Molecular Interaction Database (IntAct), Molecular INTeraction database (MINT), and biomolecular interaction network database (BIND) using the plug-in Bisogenet (Martin et al., 2010) of Cytoscape 3.7.1 software. The PPI networks of G-Rh2 putative targets and lung cancer-related targets were established and visualized by the plug-in Bisogenet of Cytoscape 3.7.1 software.

### Bioinformatic Analysis

GO analysis with the biological process, cellular component, and molecular function was carried out using the Database for Annotation, Visualization and Integrated Discovery (DAVID, https://david.ncifcrf.gov/, v6.8) (Huang et al., 2009). Functional categories were enriched within genes (FDR <0.005) and the top 10 GO functional categories were selected. KOBAS 3.0 (http://kobas.cbi.pku.edu.cn/) that assigned Kyoto Encyclopedia of Genes and Genomes (KEGG) database was used for pathway analysis (Xie et al., 2011). The significantly changed pathways which corrected *p* value <0.005 were selected and genes regulated these pathways were enriched by gene-pathway network analysis. The gene-pathway network was constructed to screen the key target genes for G-Rh2 against lung cancer.

### Cell Line and Cell Culture

The A549 lung cancer cell line was purchased from the China Center for Type Culture Collection (Wuhan, Hubei, China) and cultured in MEM supplemented with 10% FBS, 100 U/mL penicillin and 100 μg/ml streptomycin at 37°C in a humidified atmosphere with 5% CO_2_.

### Cell Viability Assay

The viability of A549 cells was measured by MTT assay. Briefly, cells were seeded into 96-well plates at 2 × 10^4^ cells/well. After adhered to the plates for overnight, cells were treated with different concentrations of G-Rh2 for 24 and 48 h. The medium was then removed and supplemented with 100 µL MTT solution for 4 h, following with 100 µL stopping buffer. The absorbance was determined at 550 nm using a microplate reader (Tecan Infinite M200 Pro, Männedorf, Switzerland).

### Cell Apoptosis Assays

The apoptosis ratio of cells was analyzed by Annexin V-FITC Apoptosis Detection Kit according to the instruction of the manufacturer. In brief, the cells treated with different concentration of G-Rh2 for 24 h were harvested and centrifuged at 1,500 rpm for 3 min to remove the medium. The precipitation was then resuspended in 100 µL 1 × binding buffer. Subsequently, the cells were stained with Annexin-FITC and PI for 15 min in the dark. After added 400 µL 1 × binding buffer, the samples were evaluated by a Accuri C6 Plus flow cytometer (Becton, Dickinson and Company, CA, United States).

### Western Blot Analysis

A total of 1 × 10^6^ A549 cells were seeded into 40 mm Petri dish and grown for overnight. Then the cells were treated with G-Rh2 for different time points. After collection, the cells were lysed by RIPA lysis buffer and the protein content was determined by Bradford reagent, using bovine serum albumin (BSA) as a standard. Total protein were separated by 20% SDS-polyacrylamide gels for 1 h at 100 V, transferred to PVDF membranes, and blocked with 5% BSA in Tris-buffered saline containing Tween 20 (1 × TTBS) for 2 h. After washed with 1 × TTBS, the PVDF membranes were then incubated with primary antibodies (1:500–1:2000) in 5% BSA at 4°C for overnight, followed by washing and incubated for 1 h with HRP-conjugated secondary anti-IgG (1:500). The bands were then visualized by the eECL Kit and photographed using Tanon 5200 Multi imaging system (Tanon, China).

### Statistical Analysis

The results have been represented as the mean ± SD. Variances among two groups were analyzed by Student’s t-test. Data analysis was completed using SPSS 20.0 (SPSS Inc., Chicago, IL, United States). *p* < 0.05 indicated significant differences.

## Results and Discussion

### Compound-Target Network Analysis

**FIGURE 2:**
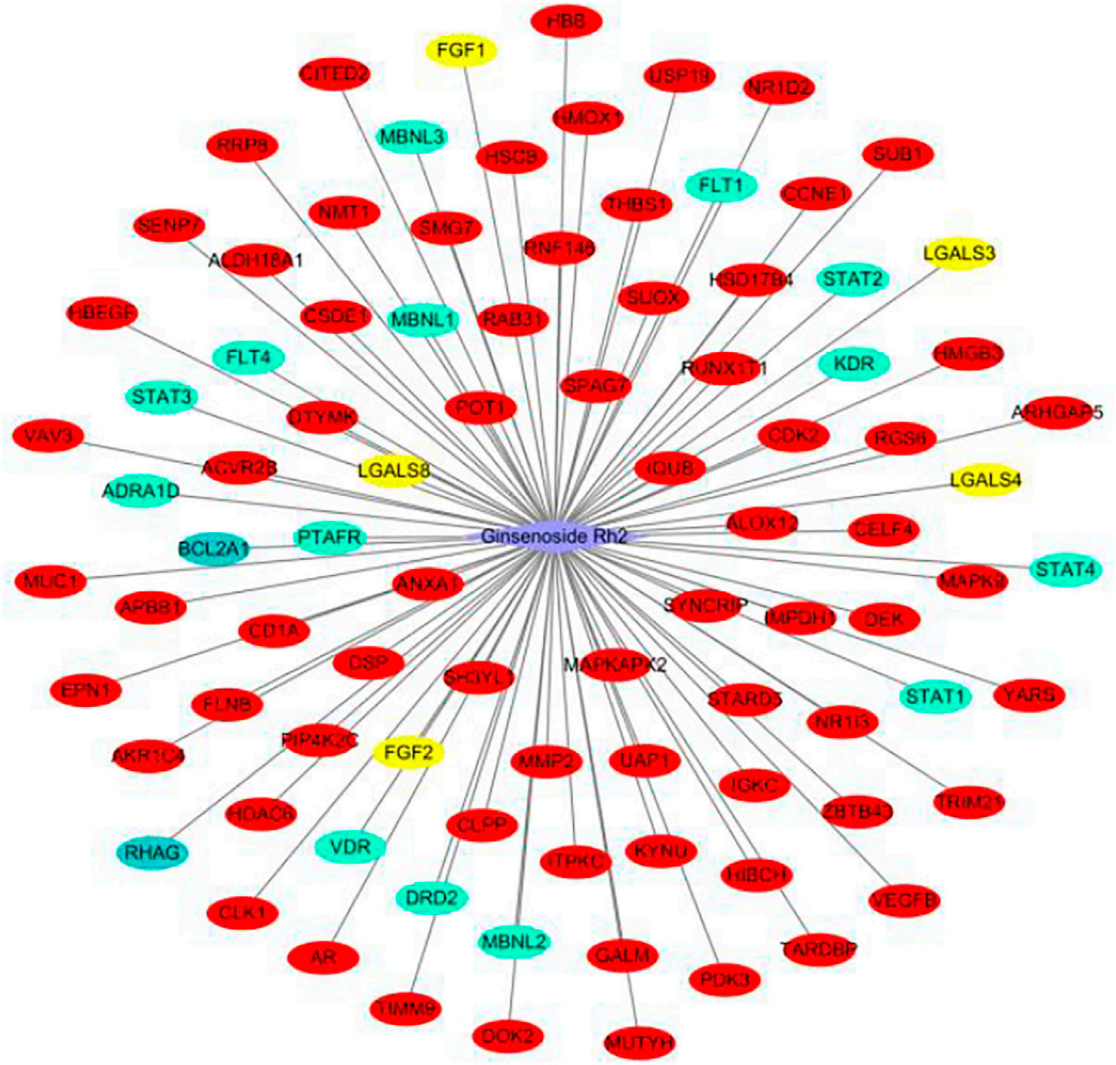
Target network of G-Rh2. Red, yellow, green, and blue ovals, are targets obtained from PharmMapper Server, Similarity ensemble approach, SwissTargetPrediction, and the STITCH database.

We evaluated the ability of G-Rh2 to inhibit the viability and induce apoptosis of A549 cells using a network pharmacology approach. The compound-target network was created by one approach. The network of G-Rh2 and its targets from PharmMapper server, SwissTargetPrediction, a similarity ensemble approach, and the STITCH database was constructed as shown in Figure 2. Ninety-one targets were obtained, among of which, 70 targets were obtained from PharmMapper server, 14 targets from SwissTargetPrediction, 5 from the similarity ensemble approach, and 2 from the STITCH database. After removing the duplicates, A total of 91 compound-targets was obtained.

### Identification of Targets for G-Rh2

**FIGURE 3:**
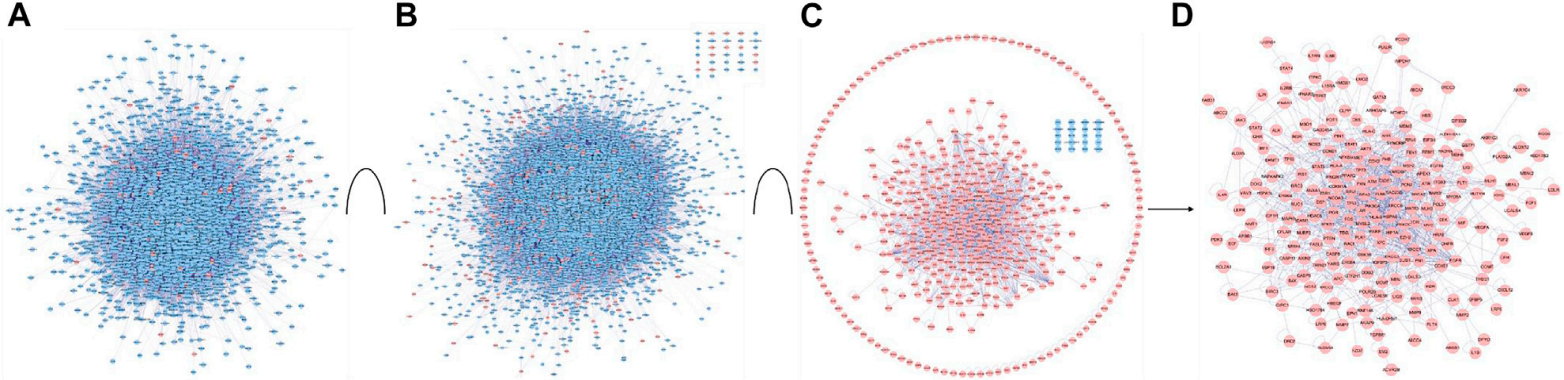
PPI network construction and analysis for targets of G-Rh2 in lung cancer. **(A)** PPI network of G-Rh2 putative targets; **(B)** PPI network of lung cancer-related targets; **(C)** Interaction network of G-Rh2 targets and lung cancer-related targets; **(D)** Merged PPI network.

Biological networked systems include functional units or protein complexes in PPI networks and disease- or drug-target genes can be analyzed according to complex network theory (Peng et al., 2018). PPIs are important in the regulation of biological systems and are the targets of an increasing number of drugs (Scott et al., 2016). PPI networks of G-Rh2 putative targets and lung cancer-related targets were structured with PPI data. The PPI network of G-Rh2 putative targets contained 3444 nodes and 86990 edges, which represented 3444 interacting proteins and 86990 interactions (Figure 3A). The PPI network of lung cancer-related targets contained 7493 interacting proteins and 179815 interactions (Figure 3B).

The interaction network of G-Rh2 comprised 91 putative targets of G-Rh2 and 431 lung cancer-related targets. The interaction network encompassed 512 interacting proteins and 1,589 interactions (Figure 3C). The structured interaction network was merged with the PPI networks of G-Rh2 putative targets and lung cancer-related targets to identify targets for G-Rh2 in lung cancer. The new network had 217 nodes and 1,028 edges (Figure 3D); thus, 217 targets for G-Rh2 in lung cancer were identified.

### GO and KEGG Pathway Enrichment Analysis

**FIGURE 4:**
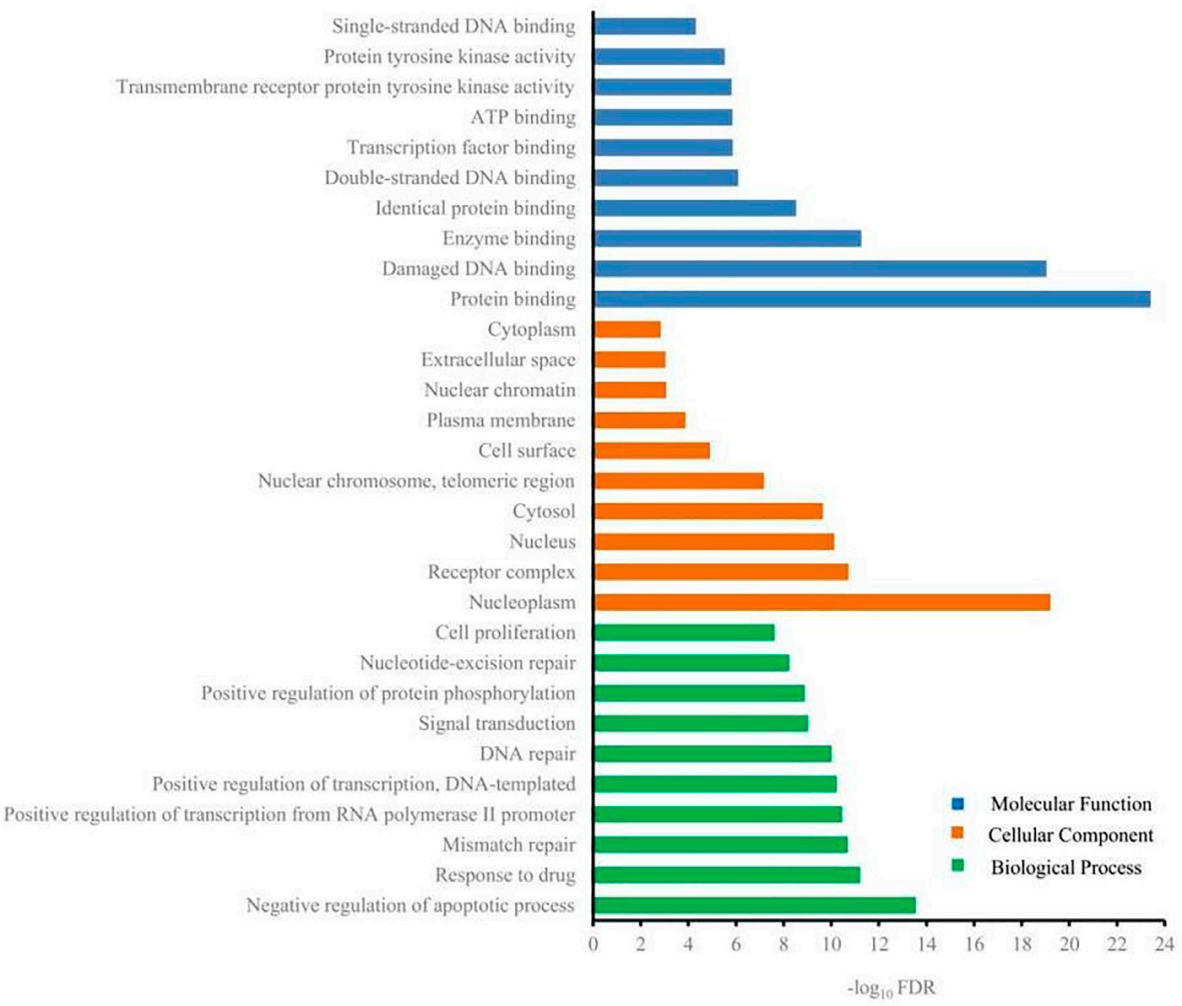
Gene ontology terms of candidate targets for G-Rh2 in lung cancer.

DAVID was used to carry out GO analysis to elucidate the function of 217 candidate targets in biological process, cellular component, and molecular function. One hundred and three GO terms with FDR <0.05 were significantly enriched: 73 in biological process, 11 in cellular component, and 19 in molecular function. The top 10 GO terms enriched in each sub-ontology are shown in Figure 4. Regulation of apoptotic process, regulation of transcription, nucleoplasm, nucleus, protein binding, and damaged DNA binding were the highly enriched GO terms. Anticancer agents activate several pathways simultaneously, positively or negatively regulating the death process (Solary et al., 2000). Regulation of apoptosis is the mechanism by which most chemotherapeutic drugs induce tumor cell death. Genetic alterations induce cancer and always result in dysregulated transcriptional programs. Almost every DNA, RNA, and protein component controlled by normal transcription is influenced by recurrent somatic mutations in tumor cells (Bradner et al., 2017). DNA binding of a new compound is an important aspect of its therapeutic potential for anticancer (Thangavel et al., 2018). Because of reversible binding or formation of covalent bonds with deoxyribonucleic acid, small DNA-interacting anticancer drugs abrogate the interaction between DNA and transcription factors in gene promoters (José, 2018). Therefore, G-Rh2 may reduce A549 cell viability by intervening in biological processes and affecting cellular components and molecular functions.

**FIGURE 5:**
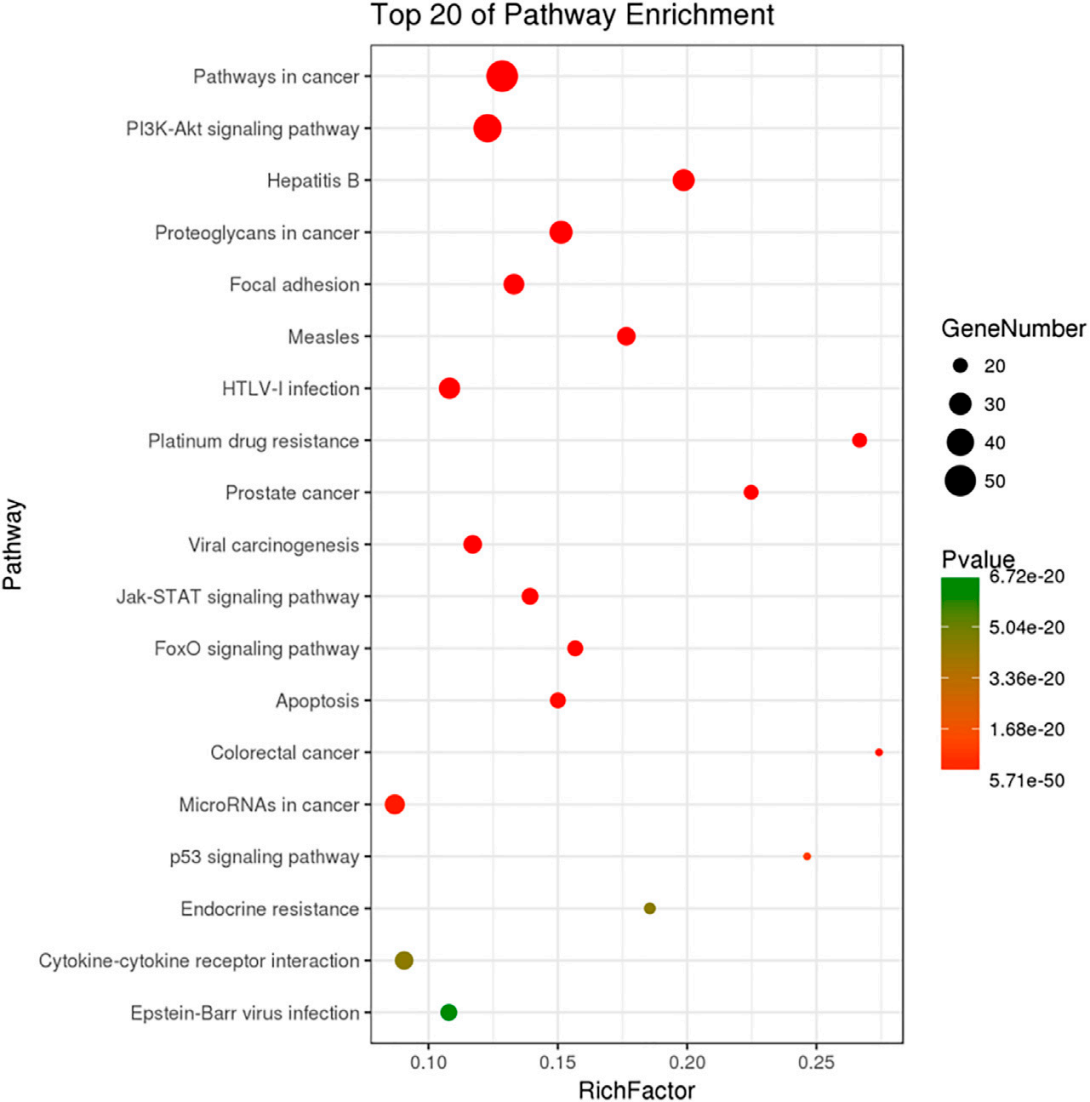
KEGG pathway enrichment of candidate targets for G-Rh2 in lung cancer.

The KEGG pathway analysis was performed by KOBAS and 143 significantly enriched pathways (corrected *p* < 0.005) including pathways in cancer, the PI3K-Akt signaling pathway, proteoglycans in cancer, focal adhesion, the Jak-STAT signaling pathway, the FoxO signaling pathway, and apoptosis were obtained. Figure 5 shows the top 20 enriched pathways. The most significantly enriched pathways were related to pathways in cancer, followed by the PI3K-Akt signaling pathway. The PI3K-Akt signaling pathway is crucial in the development of many types of tumors (Lorusso, 2016; Zhang et al., 2017). Cell proliferation, growth, cell cycle, apoptosis, and protein synthesis are regulated by the PI3K-Akt signaling pathway (Zhu et al., 2018). Also, activation of the PI3K-Akt signaling pathway promotes cancer cell proliferation, survival, and angiogenesis. The PI3K-Akt signaling pathway is activated in cancer and is a potential therapeutic target (Chen et al., 2017). Xie et al. indicated that the PI3K-Akt signaling pathway is important in lung cancer and found that ginsenoside Rg3 promoted apoptosis by inhibiting the ratio of p-PI3K/PI3K and p-Akt/Akt in A549 cells (Xie et al., 2017). Our results suggest that G-Rh2 inhibits the viability of A549 cells by inducing apoptosis *via* the PI3K-Akt signaling pathway. In addition, the Wnt/β-catenin, mTOR, VEGF, EGFR, and metabolic signaling pathways are important in lung cancer (Cho, 2013).

### Gene-Pathway Network Analysis

**FIGURE 6:**
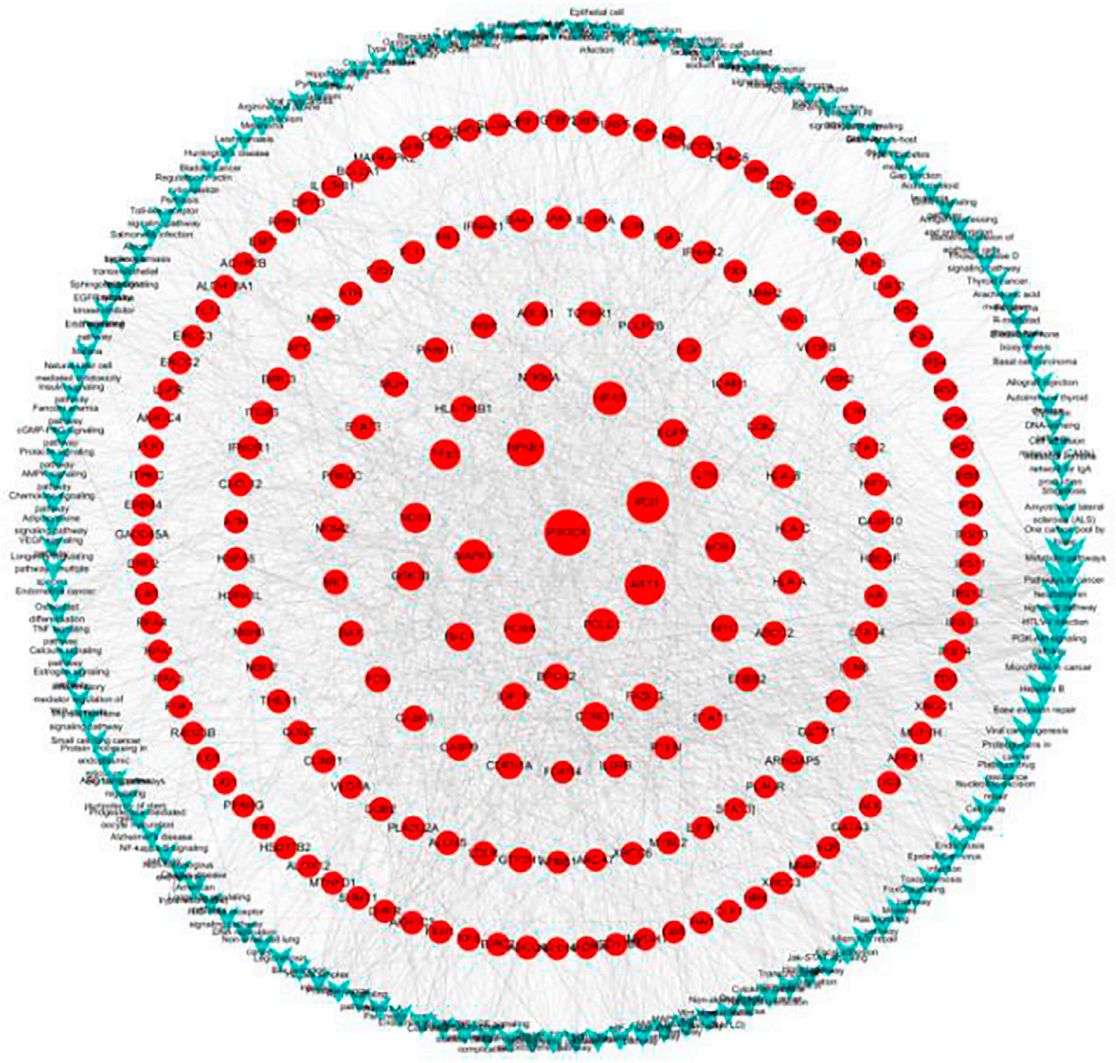
Gene-pathway network for G-Rh2 in lung cancer. Red circles, target genes; blue V-shapes, pathways. Size is proportional to betweenness centrality.

The significantly enriched pathways and genes were used to construct a gene-pathway network. As shown in Figure 6, 143 pathways and 191 genes were identified. Betweenness centrality (BC) was used to carry out the topological analysis. In the network, metabolic pathways had the highest BC, followed by pathways in cancer, the neurotrophin signaling pathway, HTLV-1 infection, and the PI3K-Akt signaling pathway. PIK3CA had the highest BC and several other genes including IRS1, AKT1, NFKB1, MAPK9, and POLD1 had larger BC. PIK3CA is one of the most commonly mutated oncogenes in human cancer and is used in the development of PI3 kinase inhibitors, which can be used as targeted therapies for cancers with these mutations (Jelovac et al., 2014). The PI3K signaling pathway is activated in cancers and is concerned with oncogenesis and cancer progression (Hennessy et al., 2005). PIK3CA mutations have been focused on as potential biomarkers of PI3K pathway activation (Ito et al., 2017). Activation of mutations and genomic amplification of the PIK3CA gene are closely related to increased PI3K activity in lung cancer (Yamamoto et al., 2008). Somatic mutations in the IA PI3K catalytic subunit p110α, encoded by PIK3CA, activate the PI3K signaling pathway (Sawa et al., 2017). IRS1 regulates many cancer-cell processes and PI3K within malignant cells (Houghton et al., 2010; Porter et al., 2013). A549 cell survival, proliferation, malignancy, and metastasis are suppressed by Akt1 knockdown (Jere et al., 2009). Also, constitutional activation of the PI3K-Akt signaling pathway can be caused by oncogenic mutations in the AKT1 gene, increasing the malignant potential of the affected cells. Our results indicate that G-Rh2 induces A549-cell apoptosis mainly by regulating the expression of PIK3CA, thus inhibiting activation of the PI3K-Akt signaling pathway. Also, regulation of IRS1, AKT1, NFKB1, MAPK9, and POLD1 may explain the G-Rh2-mediated inhibition of the viability of A549 cells.

### G-Rh2 Inhibits A549 Cell Viability and Promotes Apoptosis

**FIGURE 7:**
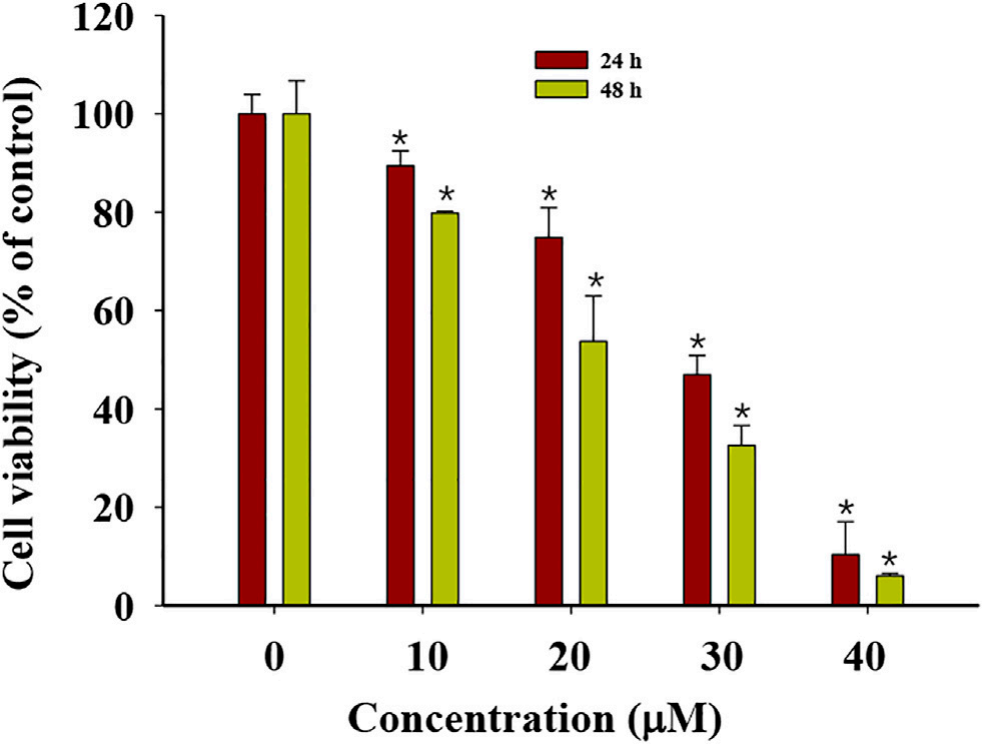
Cytotoxicity of G-Rh2 to A549 cells. Cells were treated with G-Rh2 for 24 and 48 h and viability was evaluated by MTT assay. Values are means ± SD (*n* = 3).

G-Rh2 suppresses cell proliferation, causes G1-phase arrest, enhances the activity of capase-3, and induces apoptosis in A549 cells (An et al., 2013). To validate the anti-proliferation effect of G-Rh2 on A549 cells, a MTT assay was carried out. A549 cells were treated with G-Rh2 at different concentrations for 24 h or 48 h. As shown in Figure 7, G-Rh2 significantly inhibited A549 cells proliferation in a dose-dependent manner. Based on the results of MTT assay, IC_50_ of G-Rh2 on A549 cells at 24 and 48 h were calculated respectively as 42.75 and 36.25 µM which was consist with previous report (Zhang et al., 2011).

**FIGURE 8:**
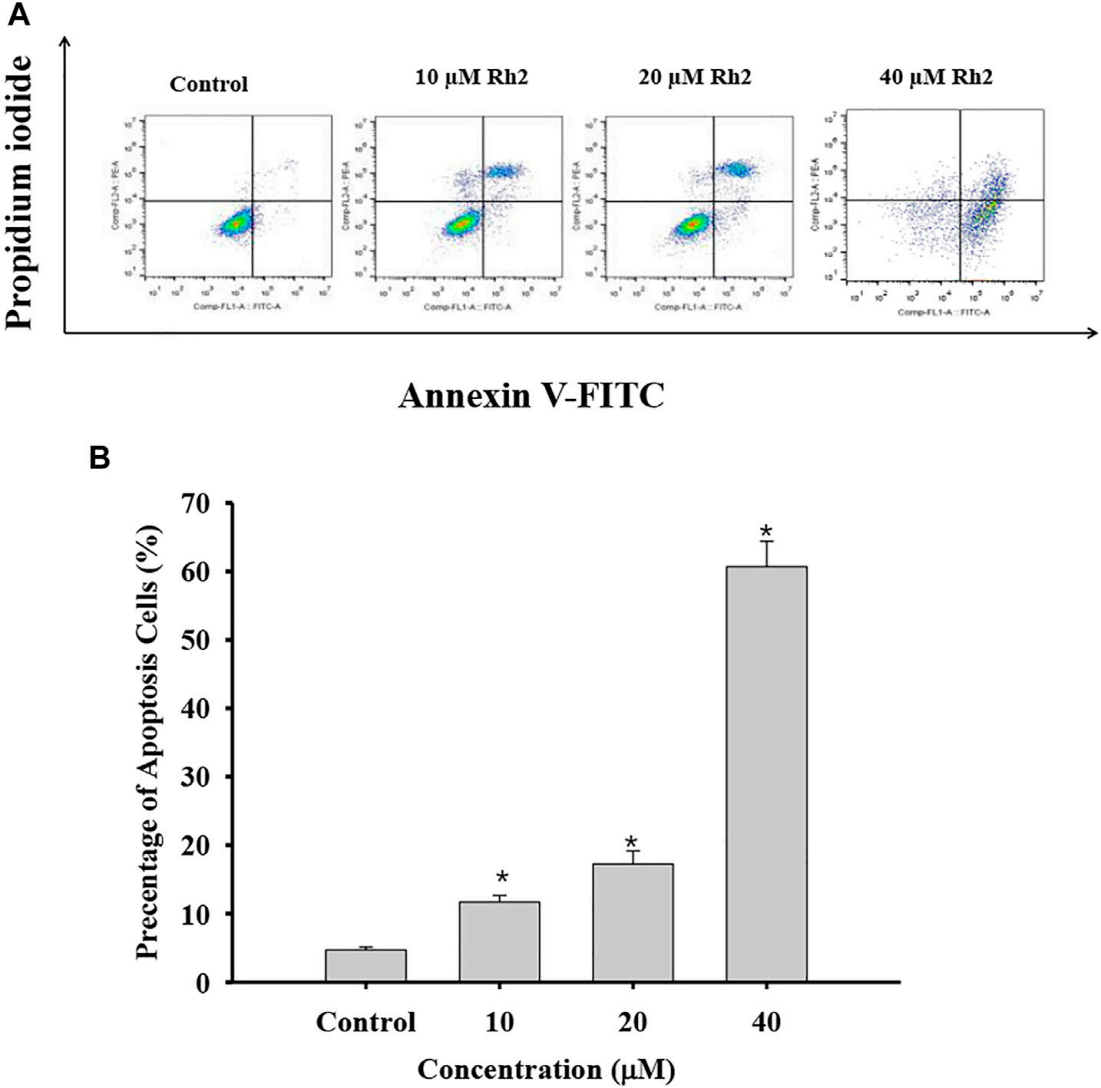
Effect of G-Rh2 on apoptosis of A549 cells. **(A)** Cells were treated with G-Rh2, and the apoptotic rate was determined by Annexin V-FITC/PI staining. **(B)** Percentage of apoptotic cells, Values are means ± SD (*n* = 3).

Apoptosis, an orderly process of programmed cell death, is central to development of cancers. It involves of the activation, expression and regulation of a series of genes, and inhibits the growth of tumor cells. Apoptosis may not the main way for the death of many cancers response to common treatments (Brown and Attardi, 2005). G-Rh2 were reported to induces apoptosis of many cancer cells, such as human epidermoid carcinoma A431cells (Park et al., 2010), human malignant melanoma A375-S2 cells (Fei et al., 2002), and hepatocellular carcinoma HepG2 cells (Zhang et al., 2019). In order to determine whether G-Rh2 can regulates A549 cell death by inducing apoptosis in this study, A549 cells treated with 10, 20, and 40 μM G-Rh2 were stained by Annexin-PI based on a flow cytometry. As shown in Figure 8, the apoptosis rate increased significantly from 4.4% in the control group to 15.92, 23.72, and 78.7% at 10, 20, and 40 μM G-Rh2, respectively. It is indicate that G-Rh2 can significantly induce A549 cells apoptosis to regulate this lung cancer cell death, and is consistent with literatures (Cheng et al., 2005; Wang et al., 2018).

**FIGURE 9:**
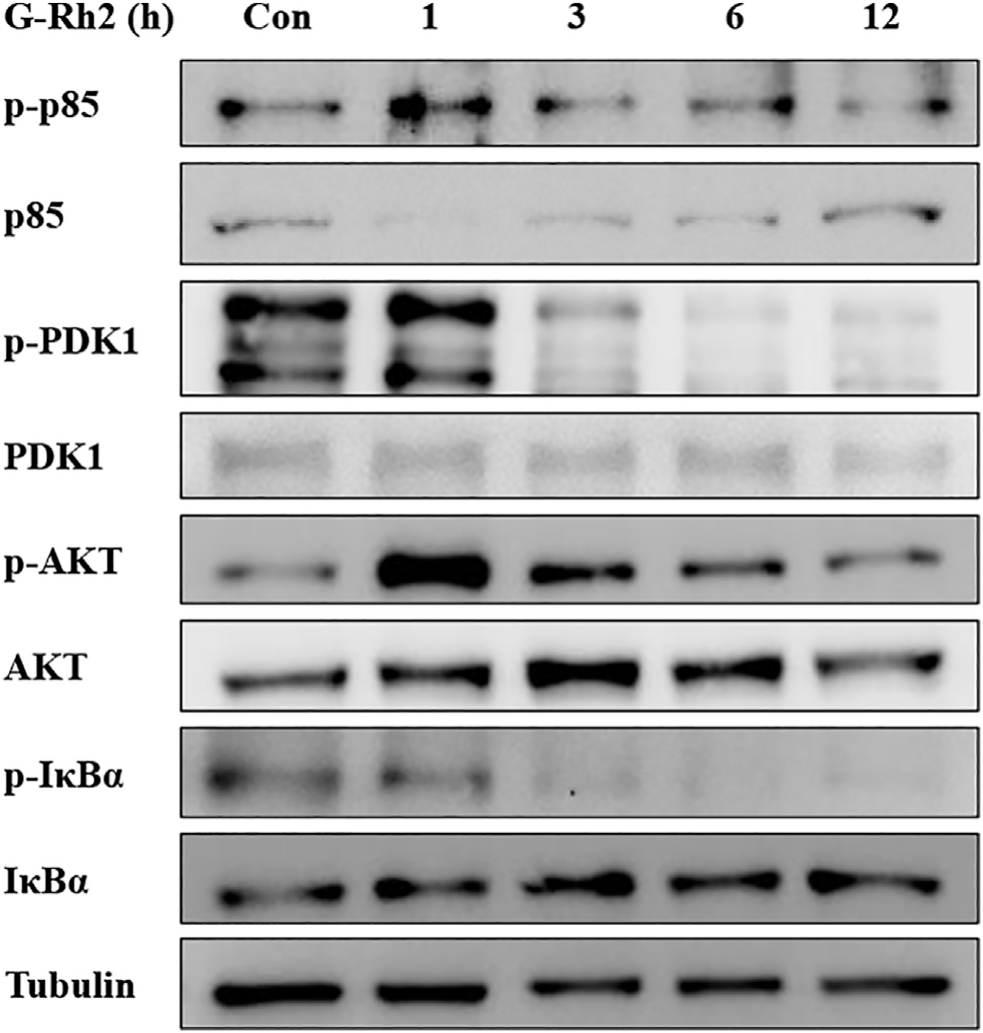
Effects of G-Rh2 on apoptosis-related proteins and their phosphorylation levels in A549 cells. Cells were treated with G-Rh2 for 0, 1, 3, 6, and 12 h. The protein levels of p85/p-p85, PDK1/p-PDK1, Akt/p-Akt and IκBa/p-IκBa were measured by Western blotting.

The PI3K/Akt signaling pathway operates by phosphorylation and dephosphorylation of the substrate-level (Sang and Li, 2007; Yu et al., 2021). PI3K, phosphoinositide 3-kinase, a lipid kinase family protein, which was constructed by two subunit including a regulatory subunit (P85) and a catalytic subunit (P110). When the growth factors binds to their receptor tyrosine kinase (RTK) or G protein-coupled receptors (GPCR), PI3K isoforms were stimulated and catalyzes the production of phosphatidylinositol-3,4,5-triphosphate (PIP3) at the cell membrane. PIP3, serves as a secondary messenger, is in turn to help the phosphorylation and activation of PDK1 and AKT. AKT is a serine/threonine kinase, once activated, it controls key cellular processes including the inhibition of apoptosis (Liu et al., 2009), and promotes cell survival by activating CREB and NF-κB activity (Park et al., 2010). Therefore, PI3K/Akt as a most commonly activated signaling pathway in human cancer, presents both an opportunity and a challenge for cancer therapy (Liu et al., 2009). In the present study, G-Rh2 was confirmed to down regulate the phosphorylation of P85, PDK1, Akt and IκBα in A549 cells by western blot analyses (Figure 9). The inactivation of P85, PDK1 and Akt by G-Rh2 treatment indicating that G-Rh2 can induces cancer cells to apoptosis *via* inhibiting the PI3K/Akt signaling pathway. Similarly, the inactivation of IκBα means G-Rh2 suppress the cell survival *via* inhibiting the NF-κB signaling pathway and to induces cancer cells death. These results are consistent with that deduced from network pharmacology analyses.

## Conclusion

The potential targets and signaling pathways of G-Rh2 on human lung cancer were predicted in this work by network pharmacology approach. According to the network pharmacology analyses results, 217 genes/proteins were predicted as potential targets for G-Rh2. Besides, bioinformatics analysis on the predicted targets revealed that over 140 pathways, of which PI3K-Akt signaling pathway is top signaling pathway involved in the underlying mechanisms of G-Rh2. Confirmatory experiments showed that G-Rh2 inhibits proliferation of human lung cancer A549 cells, and induces cells apoptosis *via* inhibiting the PI3K-Akt signaling pathway. It is consistent to the predicted results in the network pharmacology analyses.

## Data Availability

The raw data supporting the conclusion of this article will be made available by the authors, without undue reservation.
